# Longitudinal variability in mortality predicts COVID-19 deaths

**DOI:** 10.1007/s10654-021-00777-x

**Published:** 2021-07-04

**Authors:** Jon O. Lundberg, Hugo Zeberg

**Affiliations:** 1 Department of Physiology and Pharmacology, Karolinska Institutet, SE-17177 Stockholm, Sweden; 2 Department of Neuroscience, Karolinska Institutet, SE-17177 Stockholm, Sweden

**Keywords:** COVID-19, Virus, Influenza, All-cause mortality, Corona, SARS-CoV-2

## Abstract

Within Europe, death rates due to COVID-19 vary greatly, with some countries being severely hit while others to date are almost unaffected. This has created a heated debate in particular regarding how effective the different measures applied by the governments are in limiting the spread of the disease and ultimately deaths. It would be of considerable interest to pinpoint the factors that determine a country’s susceptibility to a pandemic such as COVID-19. Here we present data demonstrating that mortality due to COVID-19 in a given country could have been predicted to some extent even before the pandemic hit Europe, simply by looking at longitudinal variability of death rates in the years preceding the current outbreak. The variability in death rates during the winter influenza seasons of 2015–2019 correlates to excess mortality in 2020 during the COVID-19 outbreak (Spearman’s ρ = 0.68, 95 % CI = 0.40–0.84,* p* < 0.001). In contrast, there was no correlation with age, population density, latitude, GNP, governmental health spending, number of intensive care beds, degree of urbanization, or rates of influenza vaccination. These data suggest an intrinsic susceptibility in certain countries to excess mortality associated with viral respiratory diseases including COVID-19.

## Introduction

Seasonal fluctuations in all-cause mortality are well known with deaths typically peaking in the winter [1]. A main factor driving excess winter mortality is seasonal flu [1]. When examining mortality across countries it is clear that some countries repeatedly exhibit excess deaths during the winter flu season while others show only minimal variations [2, 3]. In 2020 the peak in excess all-cause mortality was extraordinarily large, came later, and was connected with the COVID-19 pandemic. Again, however, the higher death rates were unevenly spread throughout Europe. Here we decided to relate historical death rates 2015–2019 with the mortality rates seen in Europe during 2020 using a publically available database [3]. We find a correlation between these two parameters.

## Methods

The main data presented here were extracted from the public data base European Mortality Monitoring (EuroMomo) [3]. We defined excess deaths during 2020 as the sum of weekly Z-scores > 2 during the first 45 weeks of 2020, which correlated with reported [4] COVID-19 deaths (Spearman’s ρ = 0.81, 95 % CI = 0.63–0.91,* p* < 0001). The cutoff value of 2 was based on the fact that a Z-score − 2 to + 2 represents an approximate 95 % confidence interval. This number is also used by EuroMOMO to define the threshold for excess mortality. Z-scores are used to standardize series and enable comparison of mortality patterns between different populations or between different time periods [3]. The standard deviation is the unit of measurement of the Z-score. Here the Z-score = (number of deaths-baseline) / Standard deviation of the residuals (variation of the number of deaths around the baseline) on the part of the series used to fit the model. The Z-scores of EuroMomo are based on de-trended and de-seasonalized data, after a 2/3 power transformation as previously described [5]. Statistical dependencies were measured using Spearman’s rank correlation coefficient (ρ) and 95 % confidence intervals were constructed using the Fisher transformation of ρ [6]. Further information on how the expected mortality baseline is modelled is found in the EuroMOMO database [7].

**Fig. 1:**
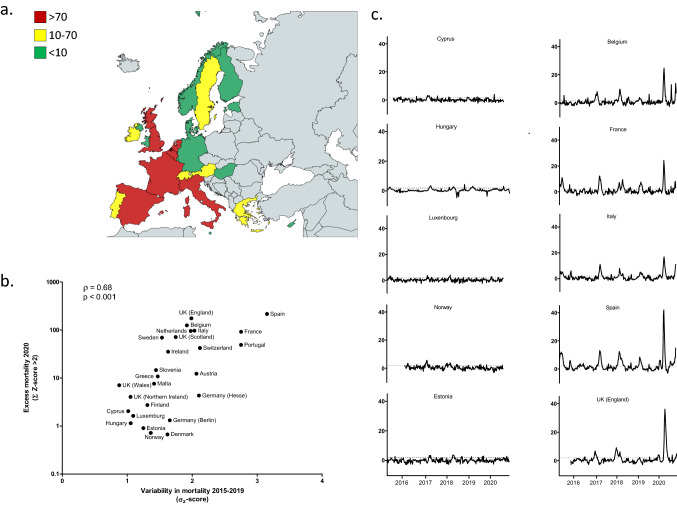
Excess mortality in Europe 2020 during the COVID-19 pandemic.** a** Map of Europe showing countries with varying degree of excess all-cause mortality during the COVID-19 outbreak in 2020, **b** variability in all-cause death rates 2015–2019 for 25 European countries plotted against the degree of excess deaths during the COVID-19 outbreak and **c** longitudinal mortality patterns (Z-scores) for 10 representative countries demonstrating low (left) or high (right) death rates during 2020. Dotted lines represent a Z-score of 2 which here is defined as the threshold for excess mortality. Countries in gray in the map are not included in the database used. For Germany two regions (Hesse, Berlin) are included in the database and calculations. Colours in the map represent different degrees of excess all-cause mortality (Z-scores, sum of 2020 Z-score > 2) with red being highest, yellow intermediate and green low

The following additional sources were used; population, age, and density [8], degree of urbanization, GNP and governmental health spendings [9], number of intensive care unit beds per capita [10], latitude [11], influenza vaccine rates [12] and a publically available map of Europe in Fig. 1 [13]. For estimation of deaths caused by COVID-19 in the studied European countries and regions up until week 45, we used data provided by Johns Hopkins CSSE [4].

## Results

Figure 1a shows the degree of excess all-cause mortality in 25 European countries during the 10 first months of 2020. The overall excess in all-cause mortality 2020 in these countries correlated with reported [4] deaths due to COVID-19 (ρ = 0.81, 95 % CI = 0.63–0.91, *p * < 0.0001). It is clear that excess mortality varies greatly within Europe in 2020. In Fig. 1b we show that countries normally experiencing fluctuating mortality exhibited higher excess mortality also in 2020 (ρ = 0.68, 95 % CI = 0.40–0.84, *p * < 0.001), while those not affected during the preceding years were spared. This pattern is clearly seen when longitudinal mortality rates (Z-scores) for some countries are exhibited (Fig. 1c). Thus, some countries, including Spain, Belgium and Italy that were severely hit by the current pandemic also displayed high excess mortality during the preceding winter influenza seasons. Conversely, other countries including Norway, Luxembourg and Estonia show hardly visible fluctuations in mortality rates over the entire study period, including 2020. When comparing the 2015–2019 Z-score variability with actual reported COVID-19 related deaths [4], we also find a correlation (ρ = 0.41, 95 % CI = 0.03–0.68, *p * < 0.05).

We also separately correlated the variability in mortality 2015–2019 with mortality during 2020 without using a cut off in the Z score. The significant correlation remained also with this method (ρ = 0.70, 95 % CI = 0.44–0.86, *p * < 0.0001).

When investigating the statistical dependencies between excess mortality 2020 (Table 1) and longitudinal variability in mortality 2015–2019 (Table 2) with other indicators, we find no significant association after controlling for multiple comparisons.

**Table 1:** Statistical dependence between some indicators and excess deaths 2020

Entity	ρ	95% CI	*p* value	N	Ref
Age (% of population + 65 years)	0.06	−0.33–0.43	0.76	26	[8]
Population density (inhabitants per Sq km)	0.19	−0.20–0.53	0.35	26	[8]
Urban Population (% of total)	0.04	−0.35–0.41	0.86	26	[9]
Latitude of the capital	−0.27	−0.59–0.12	0.18	26	[11]
Gross national product (US$) per capita	−0.09	−0.45–0.30	0.68	26	[9]
Govt. health expenditure (US$) per capita	−0.10	−0.46–0.30	0.64	26	[9]
Influenza vaccine (% coverage in 2015)	0.58	0.16–0.82	0.01	19	[12]
ICU bed per capita	−0.25	−0.58–0.14	0.21	26	[10]
Variability in Z−score (2015–2019)	0.68	0.40–0.84	0.00	26	[3]

Excess deaths 2020 defined as sum of weekly Z-scores > 2 during the first 45 weeks of 2020, which was found to correlate well with reported COVID-19 deaths (ρ = 0.81, 95% CI = 0.63–0.91, p < 0.0001). Information on influenza vaccine coverage was provided by countries recommending influenza vaccination for older people (in most countries > 65 years old) in 2014/2015. Austria, Belgium, Cyprus and Denmark did not report. Malta recommends vaccination for > 55 years old. N – the number of separate regions included in the calculation. Nominal *p* values reported in the table, with ‘Variability in Z-score’ being the only predictors surviving Bonferroni correction (p_adj_ < 0.01). The ‘Variability in Z-score’ was calculated as the standard deviation of the weekly Z-scores for periods available for each country

**Table 2:** Statistical dependence between some indicators and 2015–2019 variability in Z-score

Entity	ρ	95% CI	*p* value	N	Ref
Age (% of population + 65 years)	0.30	−0.10–0.61	0.14	26	[8]
Population density (inhabitants per Sq km)	0.17	−0.23–0.51	0.42	26	[8]
Urban Population (% of total)	0.17	−0.52–0.22	0.41	26	[9]
Latitude of the capital	−0.19	−0.53–0.21	0.35	26	[11]
Gross national product (US$) per capita	−0.13	−0.26–0.49	0.51	26	[9]
Govt. health expenditure (US$) per capita	−0.20	−0.20–0.54	0.34	26	[9]
Influenza vaccine (% coverage in 2015)	0.06	0.33–0.43	0.81	19	[12]
ICU bed per capita	−0.17	−0.22–0.52	0.40	26	[10]

The variability in Z-scores is not simply captured by any of the indicators investigated here

## Discussion

The data presented here suggest that it was possible to predict high death rates caused by COVID-19 even before the pandemic hit Europe simply by looking at fluctuations in mortality in a country during the preceding normal influenza seasons. Why then are some countries apparently unaffected by fatal respiratory viral disease year after year while others suffer considerably? Possible explanations include geographical factors [14], population demographics and density [15], genetic factors [16, 17], cultural differences along with the organization of health care and elderly nursing homes. However, we found no correlation between population age and excess mortality in 2020 and neither was there any correlation with population density, degree of urbanization, latitude, GNP, governmental health spending, available intensive care beds or rates of influenza vaccination (Tables 1 and 2). General organization and quality of health care and in particular elderly nursing homes, where a large portion of the 2020 COVID-19 related deaths originated, are more complex factors that cannot be evaluated using these data alone.

There are certain limitations to this study that deserve to be mentioned. First, a more complete picture would have been possible to obtain had we included more countries and an even longer study period. The publically available part of the EuroMOMO database dates back five years but has the advantage of a uniform reporting system for the participating countries which facilitates interpretation and comparison. Second, the mathematical formula defining the Z-scores intrinsically tends to compress mortality curves in countries with a larger natural variation in death rates, i.e., those with a smaller total population. However, if we instead directly compared the 2015–2019 Z-score variability with actual reported COVID-19 related deaths, we still observe a correlation, albeit weaker. It is also important to note that countries define and report COVID-19 deaths differently, whereas all-cause mortality is a less biased measure of the impact of a pandemic. Third, some of the indicators used such as median age are crude in nature and the study could have been improved by more fine grained indicators, e.g. capturing the full demographic profile of the country. Lastly, one should note that this study focused on the first infection wave in 2020. It is obvious that a complete picture of the situation cannot be obtained until the pandemic is fully under control.

It will be interesting to study if regional cultural differences across Europe might explain the pattern observed here. Again, such analysis will require more sophisticated analytical tools and datasets. In the case of the ongoing COVID-19 pandemic, social interactions are influenced by governmental policies ranging from milder regulations to lockdowns. The effectiveness of governmental measures in preventing the spread of infection and ultimately death is currently a matter of great debate. It seems clear to date that many countries that applied very strict measures still have experienced very high infection rates and death tolls during the current pandemic. Although the present data cannot be used to evaluate these strategies, it is noteworthy that whatever factors that drove excess mortality rates in 2020 were present already in 2015–2019, i.e., during a period when no measures were undertaken in any country. Thus our data suggest that there is an intrinsic susceptibility in certain countries to excess mortality associated with viral respiratory diseases including COVID-19.
